# Combined model integrating deep learning, radiomics, and clinical data to classify lung nodules at chest CT

**DOI:** 10.1007/s11547-023-01730-6

**Published:** 2023-11-16

**Authors:** Chia-Ying Lin, Shu-Mei Guo, Jenn-Jier James Lien, Wen-Tsen Lin, Yi-Sheng Liu, Chao-Han Lai, I-Lin Hsu, Chao-Chun Chang, Yau-Lin Tseng

**Affiliations:** 1 Department of Medical Imaging, College of Medicine, National Cheng Kung University Hospital, National Cheng Kung University, Tainan City, Taiwan, R.O.C.; 2 Department of Computer Science and Information Engineering, National Cheng Kung University, Tainan City, Taiwan, R.O.C.; 3 Department of Surgery, College of Medicine, National Cheng Kung University Hospital, National Cheng Kung University, Tainan City, Taiwan, R.O.C.; 4 Division of Thoracic Surgery, Department of Surgery, College of Medicine, National Cheng Kung University Hospital, National Cheng Kung University, No.1, University Road, Tainan City, 701 Taiwan, R.O.C.

**Keywords:** Deep learning, Radiomics, Lung nodule

## Abstract

**Objectives:**

The study aimed to develop a combined model that integrates deep learning (DL), radiomics, and clinical data to classify lung nodules into benign or malignant categories, and to further classify lung nodules into different pathological subtypes and Lung Imaging Reporting and Data System (Lung-RADS) scores.

**Materials and methods:**

The proposed model was trained, validated, and tested using three datasets: one public dataset, the Lung Nodule Analysis 2016 (LUNA16) Grand challenge dataset (*n* = 1004), and two private datasets, the Lung Nodule Received Operation (LNOP) dataset (*n* = 1027) and the Lung Nodule in Health Examination (LNHE) dataset (*n* = 1525). The proposed model used a stacked ensemble model by employing a machine learning (ML) approach with an AutoGluon-Tabular classifier. The input variables were modified 3D convolutional neural network (CNN) features, radiomics features, and clinical features. Three classification tasks were performed: Task 1: Classification of lung nodules into benign or malignant in the LUNA16 dataset; Task 2: Classification of lung nodules into different pathological subtypes; and Task 3: Classification of Lung-RADS score. Classification performance was determined based on accuracy, recall, precision, and F1-score. Ten-fold cross-validation was applied to each task.

**Results:**

The proposed model achieved high accuracy in classifying lung nodules into benign or malignant categories in LUNA 16 with an accuracy of 92.8%, as well as in classifying lung nodules into different pathological subtypes with an F1-score of 75.5% and Lung-RADS scores with an F1-score of 80.4%.

**Conclusion:**

Our proposed model provides an accurate classification of lung nodules based on the benign/malignant, different pathological subtypes, and Lung-RADS system.

**Supplementary Information:**

The online version contains supplementary material available at 10.1007/s11547-023-01730-6.

## Introduction

Non-contrast low-dose chest computed tomography (LDCT) is the standard imaging modality for lung cancer screening [1]. Based on the National Lung Screening Trial (NLST), screening LDCT can reduce mortality by 20% in the high-risk group compared with screening chest radiography [2, 3]. The use of screening chest CT is increasing, but false-positive and overdiagnosis rate are not negligible [4, 5]. In the NLST, only 3.8% of positive results were diagnosed as lung cancer [3]. In a previous study conducted at a tertiary referral center in Taiwan, we showed that 45% of resected small lung nodules of < 6 mm were benign [6].

The management of indeterminate pulmonary nodules (IPNs) is difficult [7] because most IPNs are benign [8]. Clinicians must accurately assess the risk of malignancy in order to diagnose and treat cancerous lesions without performing unnecessary tests and procedures in patients with benign nodules in a timely manner [9]. Lung-RADS, introduced by the American College of Radiology (ACR), categorizes nodules into five groups based on their risk of malignancy. Categories 1 (negative) and 2 (benign appearance) are considered negative and undergo annual screening. Categories 3 (probably benign) and 4A/4B/4X (suspicious) are considered positive and require additional evaluation before the next annual screening. Lung-RADS uses a 6 mm threshold, which reduces false positives without delaying lung cancer diagnosis compared to the 4 mm threshold used in the NLST [10, 11]. Although guidelines for nodule management are available, accurate characterization of IPNs remains tedious and subject to inter- and intra-reader variability.

Adenocarcinoma is the most common histologic subtype of lung cancer [12]. Atypical adenomatous hyperplasia (AAH) may be a precursor lesion of adenocarcinoma [13]. The invasiveness of lung adenocarcinoma is assessed by a multidisciplinary classification [10], categorizing it as adenocarcinoma in situ (AIS), minimally invasive adenocarcinoma (MIA), or invasive adenocarcinoma (IA). Given the central role of diagnosis in treatment and prognosis, invasiveness has a significant impact on survival [14, 15]. Improving the prediction of invasiveness by chest CT offers significant clinical benefit to patients with lung cancer.

Computer-aided detection (CAD) in chest CT has long been recognized for its ability to improve sensitivity in nodule detection [16, 17]. Recent breakthroughs in deep learning (DL) for medical imaging have expanded its capabilities to include automatic nodule segmentation [18], classification [19], nodule measurement, and malignancy risk assessment [20]. However, most of the previous studies were conducted under conditions that differ from real-world practice and often selected for disease prevalence and dichotomized distribution. In addition, current CAD cannot predict lung nodule pathology preoperatively. Therefore, there is a significant need for studies that evaluate artificial intelligence (AI) models in real-world populations and provide preoperative prediction of lung nodule pathology to guide clinical decision making.

Lung nodules can be classified using two basic approaches: (1) the radiomic feature extraction from chest CT scans, either 2D or 3D [21, 22], and (2) convolutional neural networks (CNN) [23, 24]. Many recent studies have used these tools to predict the invasiveness of lung nodules [23, 25–30]. The radiomics approach requires an appropriate lung segmentation and feature extraction algorithm to classify the tumor, while CNN does not need such an algorithm, but requires a huge dataset [20]. In this study, we aimed to investigate the diagnostic performance of our proposed AI model in open dataset, and private dataset (both surgery and health checkup participants). The purpose of our study was to investigate whether our proposed combined AI model (integrating DL, radiomics, and clinical data) could improve the classification of lung nodules into benign/malignant, histological types and Lung-RADS categorization.

## Materials and methods

This retrospective study was approved by the institutional review board of National Cheng Kung University Hospital (A-ER-108–359) and the requirement for written informed consent was waived because the data were analyzed retrospectively and anonymously.

### Datasets

The following three datasets were used:

1. Lung Nodule Analysis 2016 (LUNA16) Grand Challenge dataset [31]: This is a publicly available dataset containing1186 lung nodules from 888 patients.
2. Lung Nodule Received Operation (LNOP) dataset: It includes 1027 lung nodules from 708 patients who underwent surgical resection with histopathological diagnosis at the National Cheng Kung University Hospital (NCKUH) between December 2018 and December 2021.
3. Lung Nodule in Health Examination (LNHE) dataset: It includes 1525 lung nodules from 653 patients, which were found during a healthy examination between January 2019 and December 2021 at the NCKUH.

**Fig. 1:**
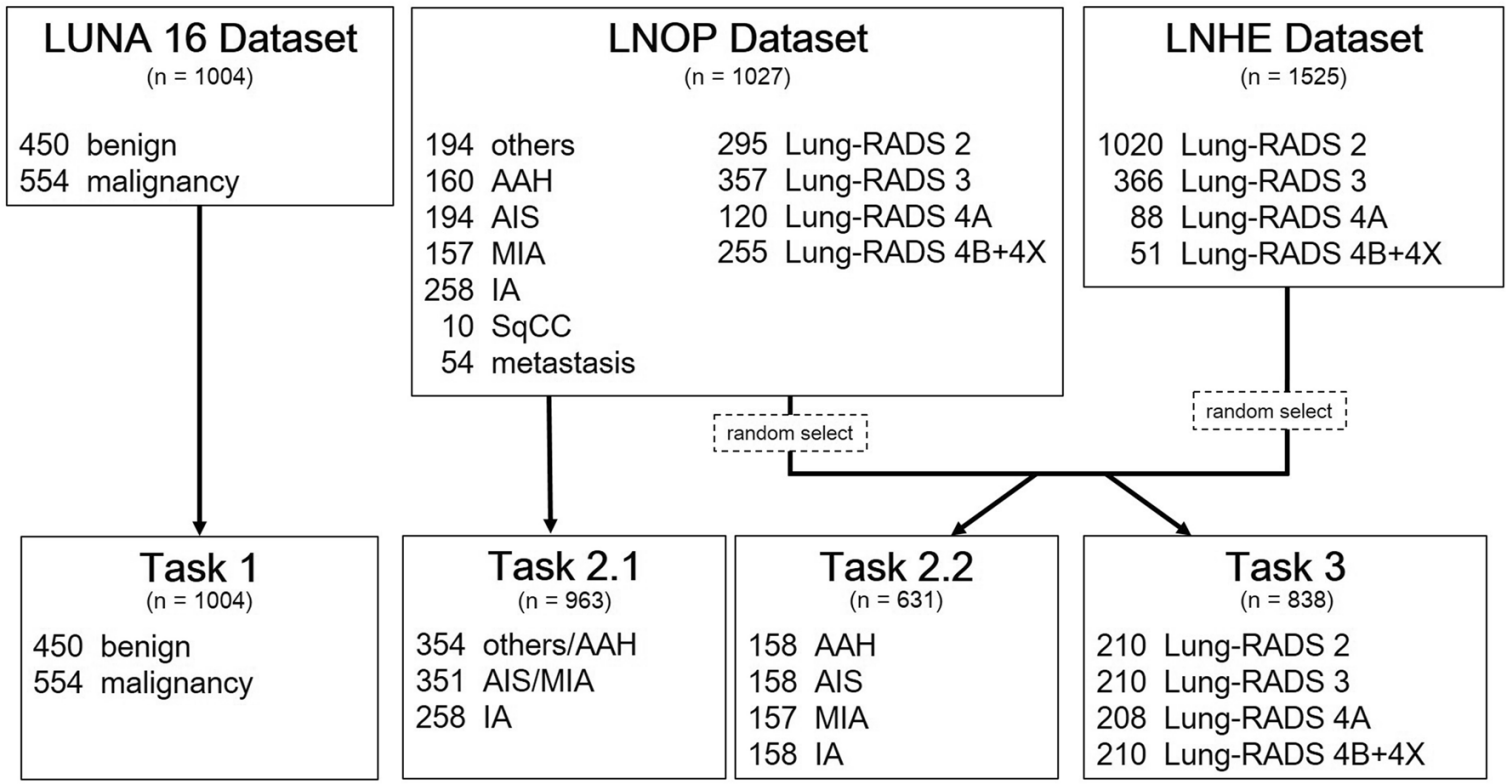
Flowchart of the analysis cohort

Figure 1 shows how the datasets were partitioned for each task. Ten-fold cross-validation was applied to each task. The LNOP dataset also includes clinical information that have been observed to be associated with lung cancer and tumor phenotype [32–36], such as age, sex, smoking history (defined as positive smoking history regardless of whether the patient is an active smoker or has quit smoking) and the presence of a family history of lung cancer in first-degree relatives.

### Classification tasks

We performed three classification tasks in this study:

1. Task 1: Classification of lung nodules as benign or malignant in the LUNA16 dataset.
2. Task 2.1: Three-class classification of
  (i) IA
  (ii) MIA + AIS
  (iii) AAH + other benign lesions.
  Task 2.2: Four class classification of AAH, AIS, MIA, an d IA.
3. Task 3: Four-class classification of Lung-RADS score: 2, 3, 4A, 4B + 4X.

Task 2.1 was designed to resemble the real clinical scenario based on treatment strategies: (i) IA has the worst prognosis, and lobectomy is often recommended [37], (ii) MIA + AIS have almost 100% survival probability, and limited resections are suggested [37, 38], (iii) AAH + other benign lesions required conservative treatment or follow-up. Task 2.2 provided four-class classification of adenocarcinoma spectrum lesions from pre-invasive to invasive lesions into AAH, AIS, MIA, and IA. Only the LNOP dataset was used in Task 2, whereas the LNOP and LNHE datasets were used in Task 3. Squamous cell carcinoma (SqCC) and metastasis were excluded in Task 2.1 and Task 2.2 due to their rarity in the lung screening program. To balance the data, only partial data were used in Tasks 2.2 and 3.

### Image acquisition

In the LUNA16 dataset, the size of all the nodules was greater than 3 mm and slice thickness was less than 2.5 mm. In the LNOP and LNHE datasets, all CT images were acquired using the Siemens SOMATOM Definition Flash, Siemens SOMATOM Definition AS, SOMATOM Definition Edge, and GE Optima CT660. The CT protocols were as follows: 120 kVp; tube current, 150–200 mA with automatic tube current modulation in the LNOP dataset and 30 mA in the LNHE dataset. The slice thickness ranged from 0.625 to 1.5 mm, and the image size was 512 × 512 pixels.

### Image preprocessing

The image preprocessing module included (1) voxel resampling, (2) cropping, and (3) Hounsfield unit conversion. Because the voxel spacing of each CT image might be different, we resampled the voxel spacing of all CT images and masks to the smallest spacing value in the dataset (0.48 × 0.48 × 0.625 mm3). The 3D CT images were then cropped to the size of 32 × 32 × 32 as the input to the DL model. Finally, the Hounsfield unit in the range between -1024 and 400 was converted to a decimal between 0 and 1 and stored in the single precision floating point format. This is a normalization of the input data for the neural network.

### Radiomic feature extraction

**Fig. 2:**
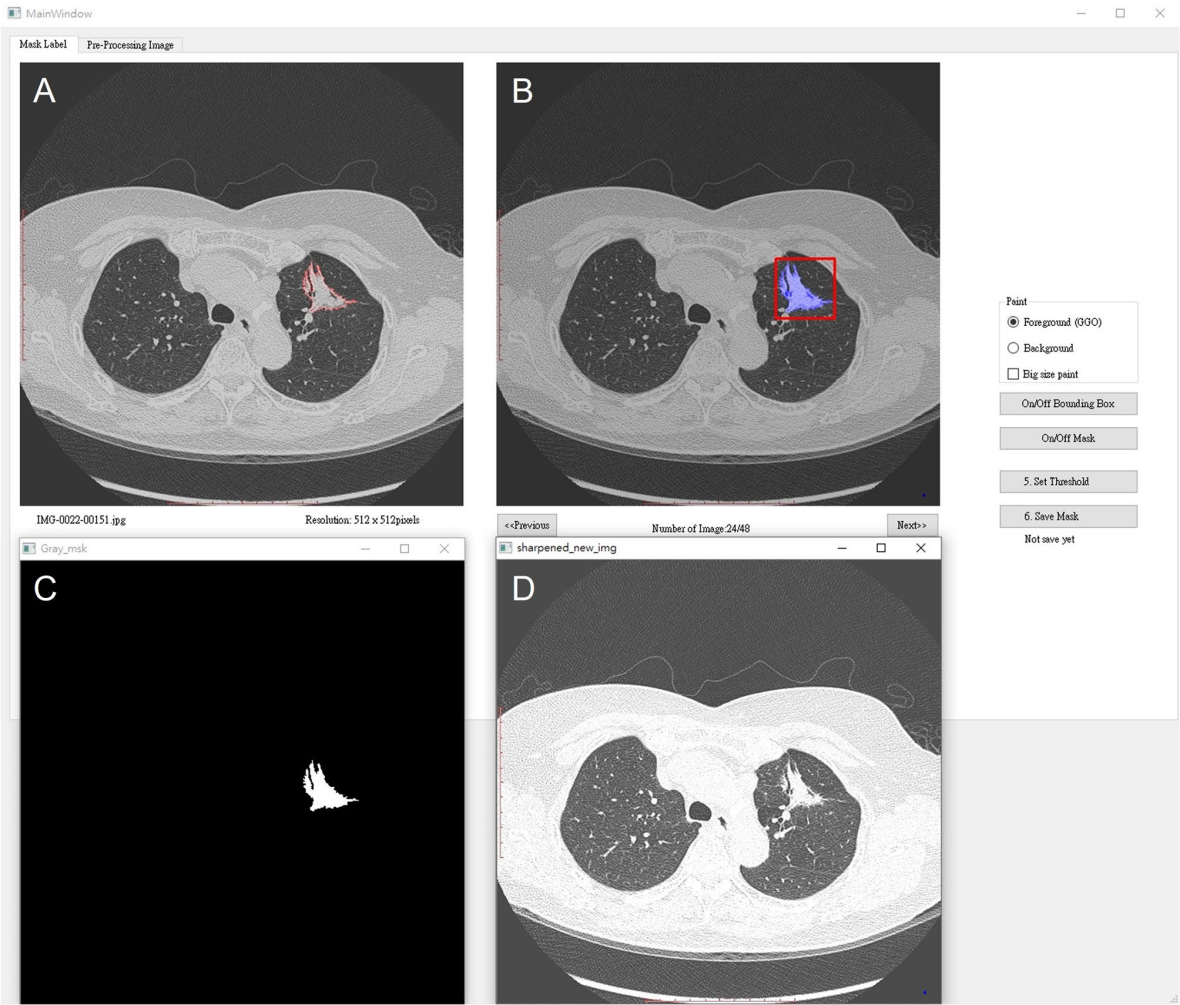
Graphical user interface for the lung nodule segmentation. **A** Lung nodule segmentation is manually performed by a thoracic radiologist. **B** A bounding box created from a segmentation mask automatically. **C** A segmentation mask is provided automatically. **D** The original non-enhanced axial CT images in lung window setting shows an irregular left upper lobe (LUL) mass

Contours defining the 3D tumor region of interest were manually drawn slice by slice on axial images after a consensus was reached between the thoracic radiologist (C.Y.L., with 10 years of experience) and the thoracic surgeon (C.C.C., with 10 years of experience). The thoracic radiologist blinded to the clinicopathologic data performed tumor segmentation by using graphical user interface (GUI) written in Python. Tumor delineation was performed in a lung window setting to highlight lung structures on the axial CT plane, including bronchi, blood vessels, and vacuoles within the nodules and excluding irrelevant normal lung tissue, mediastinal structures, and chest wall, as shown in Fig. 2.

A total of 1319 radiomic features were extracted by using Pyradiomics [39] with two image filters, the Laplacian of Gaussian (LoG) filter and the wavelet filter to highlight specific features [41], and six feature families, including shape features, first-order statistics, and texture features (gray level co-occurrence matrix (GLCM), gray level run length matrix (GLRLM), gray level size zone matrix (GLSZM), and gray level dependence matrix (GLDM)) [40–42].

### DL model

The DL models were derived from a modified 3D CNN, NASLung [43]. To improve the model performance, we made two modifications. First, we replaced the convolutional block attention module (CBAM) with a coordinate attention (CA) block to ensure that the model efficiently captures long-range features with accurate location information [44, 45]. Second, the residual block in NASLung was adapted to be applicable to lung nodule classification, inspiration by the dilated residual dense block (DRDB) [46]. The training environment and strategy were detailed in Appendix S1.

### Development and combination of models for lesion classification

**Fig. 3:**
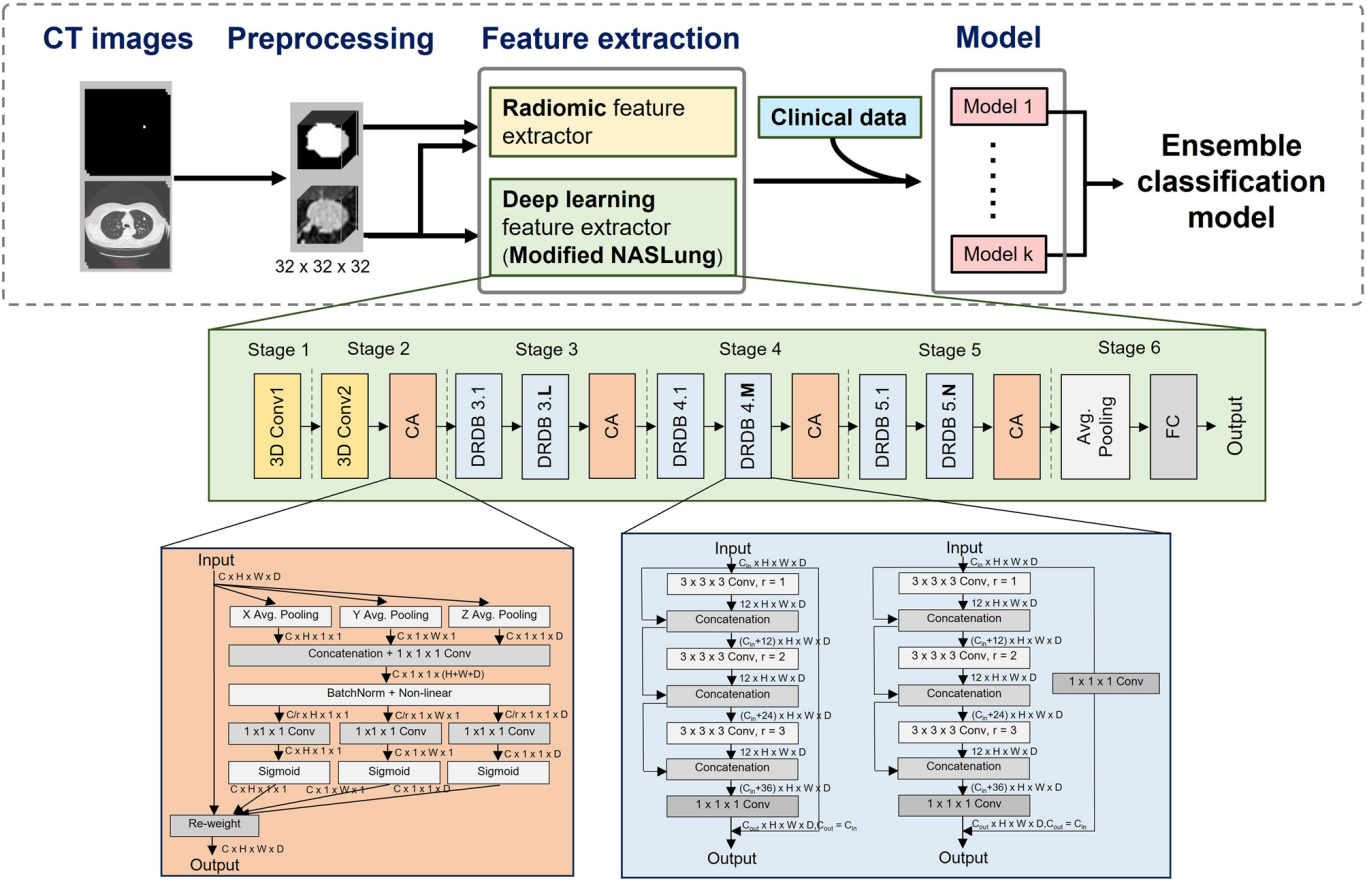
Data processing pipeline. The parallel radiomics and DL model encodes the input images to features which be combine with clinical parameters. Then the combined features be classified by an ensemble classification model. The methods denoted as L, M, and N in modified NASLung architecture were determined based on the original NASLung framework

The global framework is shown in Fig. 3. To combine the DL and radiomics models, we applied a stacked ensemble model using a machine learning (ML) approach with the AutoGluon-Tabular classifier [42]. Autogluon is an open-source automated ML library developed by the Amazon Web Services (AWS). It serves as a framework for automating ML tasks, allowing users to automatically select and train ML models. In AutoGluon-Tabular, it integrates several basic ML models, including neural networks, LightGBM boosted trees, CatBoost boosted trees, Random Forests, Extremely Randomized Trees, and K-Nearest Neighbors algorithm. For each ML model selected, Autogluon optimizes the training process by automating hyperparameter tuning, achieving superior performance by eliminating manual iteration through hyperparameter configurations. Additionally, it uses multi-layer stacking strategies to enhance prediction performance. The ensemble model provided a probability of malignancy (Task 1), a histopathology result (Task 2.1 and 2.2), and a Lung-RADS score (Task 3). To test whether the clinical features had any additional predictive value for histopathologic subtype classification, the ensemble model was retrained after the addition of clinical features for Task 2.1 and 2.2.

### Radiologist reading

Target lesions were independently identified and classified according to Lung-RADS version 1.1 by a thoracic radiologist (C.Y.L., with 10 years of experience) who was blinded to all patient clinical and demographic information. In addition, the imaging and histopathologic results of each patient in the LNOP dataset were reviewed by a multidisciplinary thoracic tumor board as a standard of care.

#### Statistics

Continuous variables were reported as the mean ± standard deviation, and categorical variables were reported as *n* (%). In the ten-fold cross-validation, the results were presented as mean accuracies. To verify the performance of the different classification models, we calculated the accuracy, macro-recall, macro-precision, and macro-F1 scores. Accuracy is the ratio of the correctly classified samples to the total samples. The precision is the ratio of the true positive results to all positive results, and the average of the precision of each sample label is the macro-precision. The recall is defined as true-positive results divided by the sum of true-positive and false-negative results, and the mean value of the recall of each sample label is the macro-recall. The macro-F1 score is the harmonic mean of the macro-precision and macro-recall.

## Results

### Dataset characteristics

**Fig. 4:**
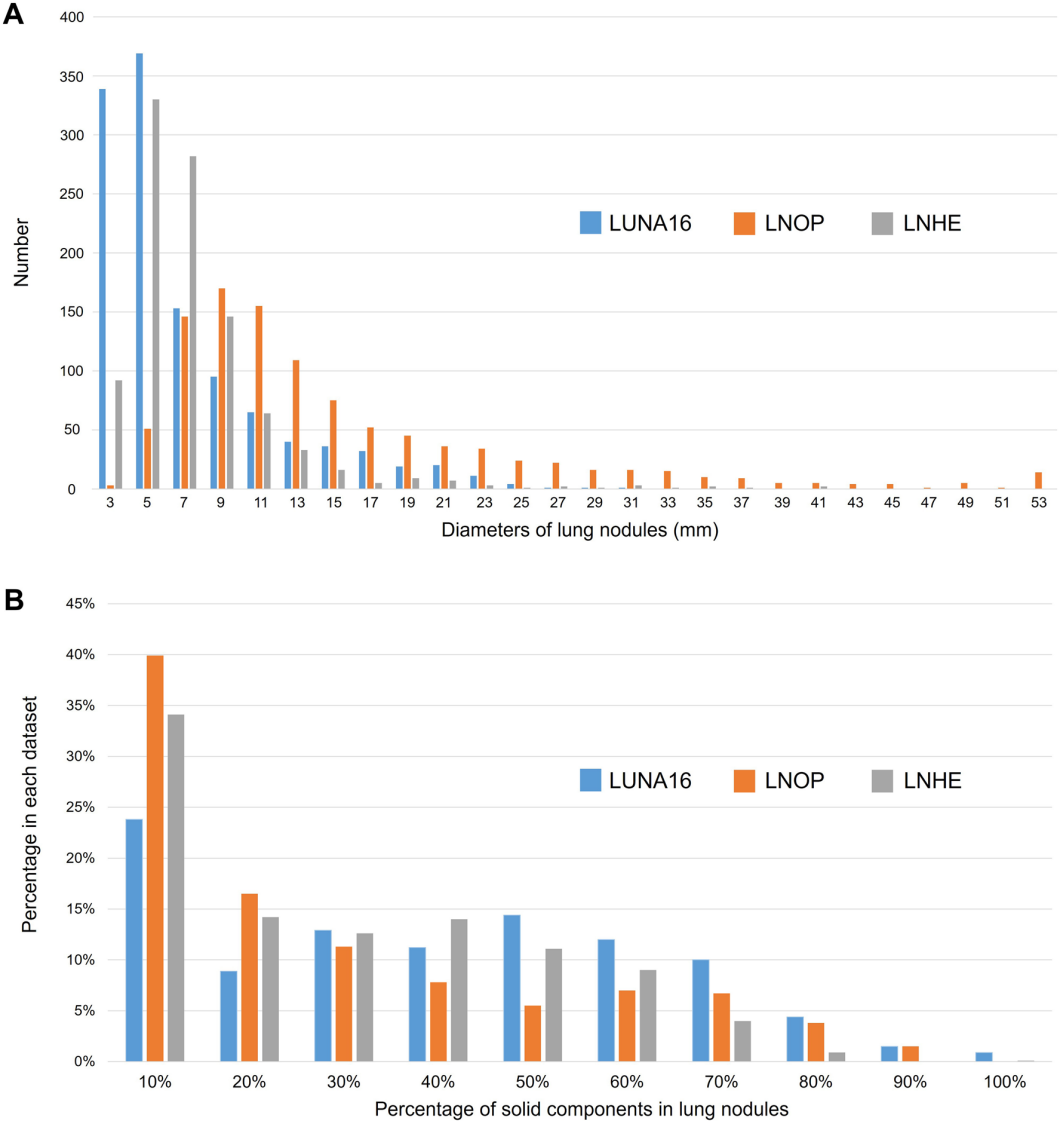
Dataset component analysis. **A** Nodule diameters: Diameters of lung nodules in LUNA16, LNOP, and LNHE datasets. **B** Solid components: Proportions of solid components in lung nodules from LUNA16, LNOP, and LNHE datasets

Figure 4 shows the 3D diameter distribution and percentage of solid components in lung nodules from the LUNA16, LNOP, and LNHE datasets. Clinical and pathologic characteristics are detailed in Table 1. Females comprised the majority of patients in both the LNOP (64.5%) and LNHE (53.4%) groups. The mean age of the patients was 59.6 years in the LNOP group and 54.7 years in the LNHE group. Among the patients in the LNOP group, 27.8% had a smoking history, while 16.0% had a family history of lung cancer in first-degree relatives. IA was the most common pathology among patients in the LNOP group, accounting for 25.1% of all malignancies. According to Lung-RADS, 36.5% of the patients in the LNOP group were classified as 4A + 4B + 4X. In contrast, because the patients in the LNHE group were all asymptomatic individuals undergoing health screening, only 9.1% were classified as 4A + 4B + 4X.

**Table 1:** The clinical and pathological characteristics of LNOP and LNHE datasets

	LNOP		LNHE	
	Patient (n = 708)	Nodule (*n* = 1027)	Patient (*n* = 653)	Nodule (*n* = 1525)
Sex (male)	251 (35.5%)	339 (33.0%)	304 (46.6%)	632 (41.4%)
Age*	59.6 ± 10.7	59.1 ± 11.2	54.7 ± 12.6	55.5 ± 9.7
Smoking	197 (27.8%)	256 (24.9%)	–	–
Family history	113 (16.0%)	125 (12.2%)	–	–
Pathology				
AAH	–	160 (15.6%)	–	–
AIS	–	194 (18.9%)	–	–
MIA	–	157 (15.3%)	–	–
IA	–	258 (25.1%)	–	–
SqCC	–	10 (1.0%)	–	–
Metastasis	–	54 (5.3%)	–	–
Others	–	194 (18.9%)	–	–
Lung-RADS				
2	–	295 (28.7%)	–	1020 (66.9%)
3	–	357 (34.8%)	–	366 (24.0%)
4A	–	120 (11.7%)	–	88 (5.8%)
4B + 4X	–	255 (24.8%)	–	51 (3.3%)

Note.—Unless otherwise indicated, data are numbers of patients, and data in parentheses are percentages

^*^Data are mean ± standard deviation

In terms of 3D diameter, the majority of nodules in the LUNA16 and LNHE datasets were smaller than 30 mm, while LNOP had a wider distribution with a maximum of 53 mm. The wider distribution of nodule sizes in the LNOP dataset may be attributed to the fact that nodules deemed suitable for surgical removal were more likely to be malignant based on the subjective judgment of the clinicians. Regarding the solid component, the LUNA16 dataset had a higher proportion of solid components compared to the LNOP and LNHE datasets. This discrepancy may be due to the fact that the LNOP and LNHE datasets consisted primarily of individuals of Asian descent, who had a higher prevalence of ground-glass opacities.

### Classification of lesions as benign or malignant

**Fig. 5:**
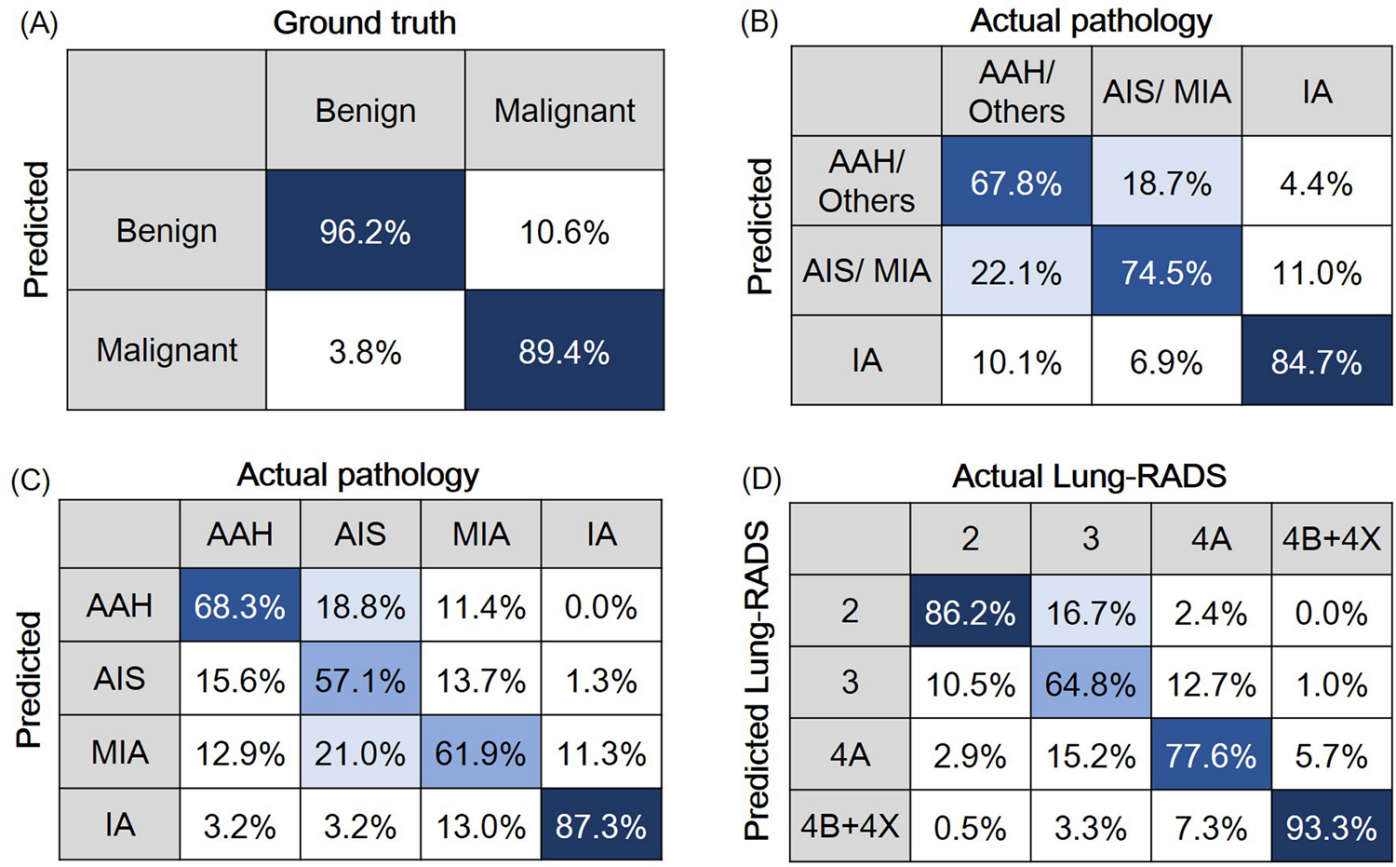
The confusion matrices of predictive performance of combined model. **A** The distinguishing ability of combined model in classifying benign and malignancy with LUNA 16 dataset. **B**, **C** Combined model’s prediction of pathology. **D** Combined model’s prediction of Lung-RADS

Our classification model had an accuracy of 92.80% in ten-fold cross-validation and an F1-score of 92.16% (Table 2). The confusion matrix of the LUNA16 classification is shown in Fig. 5A. The accuracy of the benign lesion was 96.17% and the accuracy of the malignant lesion was 89.43%.

**Table 2:** Model performances for different tasks

Task	Accuracy (%)	F1-score (%)
Task 1		
Our method	92.80^#^	92.16
Zhang et al. (2022)	92.75^#^	
Jiang et al. (2021)	90.77*	
Xia et al. (2021)	91.90^#^	
Zhu et al. (2018)	90.44*	
Task 2.1	74.76	75.45
Others/AAH		71.52
AIS/MIA		72.76
IA		81.49
Task 2.2	68.52	68.70
AAH		68.67
AIS		59.97
MIA		59.89
IA		84.53
Task 3	80.48	80.38
Lung-RADS 2		83.91
Lung-RADS 3		68.63
Lung-RADS 4A		76.81
Lung-RADS 4B + 4X		91.39

^*^ Fivefold cross-validation accuracy. ^#^ Tenfold cross-validation accuracy

In the ablation study for Task 1, we evaluated the effectiveness of modified NASLung and the incorporation of radiomics features. In the DL model, we incrementally added image preprocessing, replaced the CBAM and residual block in the original NASLung with CA and DRDB, and used the AutoGluon-Tabular classifier. This ultimately increased the accuracy from 88.78% to 92.21% and increased the F1-score from 87.92% to 91.61%. In the radiomics model, after image preprocessing and applying the AutoGluon-Tabular classifier, using all radiomics features showed the best result with an accuracy of 90.75%, F1-score 89.86. However, the result was inferior to the DL model. The combination of DL model and radiomics model showed the best result, with an accuracy of 92.80%, F1-score 92.16% (Table 3).

**Table 3:** Ablation tests on classification performance of 3D CNN modification

	Image preprocessing	CA	DRDB	Redoing NAS	Image Type			Feature Class			AutoGluon	Accuracy (%)	F1-score (%)	Precision (%)	Recall (%)
					Original	LoG	Wavelet	Shape descriptors	First order statics	Texture					
DL model															
	Original NASLung											88.78	87.92	91.98	84.25
	✔											89.53	88.84	91.36	86.57
	✔	✔										90.40	89.75	92.42	87.67
	✔	✔	✔									91.62	90.94	93.01	88.46
	✔	✔	✔	✔								92.14	91.54	94.08	88.57
	✔	✔	✔	✔							✔	92.21	91.61	94.51	88.65
Radiomics model															
	✔				✔			✔	✔	✔	✔	88.20	87.01	93.31	81.99
	✔					✔			✔	✔	✔	89.24	88.35	91.74	85.49
	✔						✔		✔	✔	✔	90.53	89.85	93.45	86.70
	✔				✔			✔			✔	85.70	84.29	90.34	79.49
	✔				✔	✔	✔		✔		✔	89.55	89.03	90.36	87.85
	✔				✔	✔	✔			✔	✔	90.21	89.40	92.59	86.58
	✔				✔	✔	✔	✔	✔	✔	✔	90.75	89.86	95.00	85.45
DL + radiomics model															
	✔	✔	✔	✔	✔	✔	✔	✔	✔	✔	✔	92.80	92.16	95.22	89.43

### Three-class classification of IA, MIA + AIS, AAH + other benign lesions

The F1-score and the overall accuracy of Task 2.1 were 75.45% and 74.76%, respectively (Table 2). The confusion matrix of Task 2.1 is shown in Fig. 5B. The accuracy of IA, AIS/MIA, and AAH/others was 84.70%, 74.45% and 67.82%, respectively.

In the ablation study for Task 2.1, we investigated the effect of clinical features on the prediction of malignancy in lung nodules. After incorporating four clinical features, namely smoking history, family history, age, and sex, we observed an increase in the accuracy from 72.87% to 74.76% and in the macro F1-score from 73.57% to 75.45% (Table S1).

### Four-class classification of AAH, AIS, MIA, and IA

The F1-score and the overall accuracy of Task 2.2 were 68.70% and 68.52%, respectively (Table 2). The confusion matrix of Task 2.2 is shown in Fig. 5C. The accuracies of IA, MIA, AIS, and AAH were 87.33%, 61.88%, 57.06% and 68.27%, respectively. The model performed better in distinguishing AAH from IA, but showed poorer performance in distinguishing AIS from MIA.

### Four-class classification of lung-RADS score 2, 3, 4A, 4B + 4X

The F1-score and the overall accuracy of Task 3 were 80.38% and 80.48%, respectively (Table 2). The confusion matrix of Task 3 is shown in Fig. 5D. The accuracies of Lung-RADS 4B + 4X, 4A, 3, and 2 were 93.34%, 77.56%, 64.76% and 86.19%, respectively. In addition, the relationship between final pathology and Lung-RADS score in the LNOP dataset was presented in Table S2. The LNOP dataset showed a higher risk of malignancy when using Lung-RADS categorization as a reference (Lung-RADS score 2: 39.8%, score 3: 70.9%, score 4A: 77.3%, score 4B + 4X: 80%).

## Discussion

We proposed a combined model to classify lung nodules using radiomics and DL. In addition, we evaluated the added value of clinical features to classify different pathologic subtypes of lung nodule. Our fusion model achieved high predictive accuracy and outperformed all other single method-based models in the LUNA16 dataset after module modification. We achieved F1-scores of 75.45% and 68.7% in three- and four-class classifications, respectively, when predicting pathological subtypes using private datasets. Classification of the Lung-RADS score gave an accuracy of 80.38%. Our proposed model demonstrated high accuracy in classifying lung nodules as benign or malignant, as well as in classifying lung nodules into different pathological subtypes and Lung-RADS scores.

**Fig. 6:**
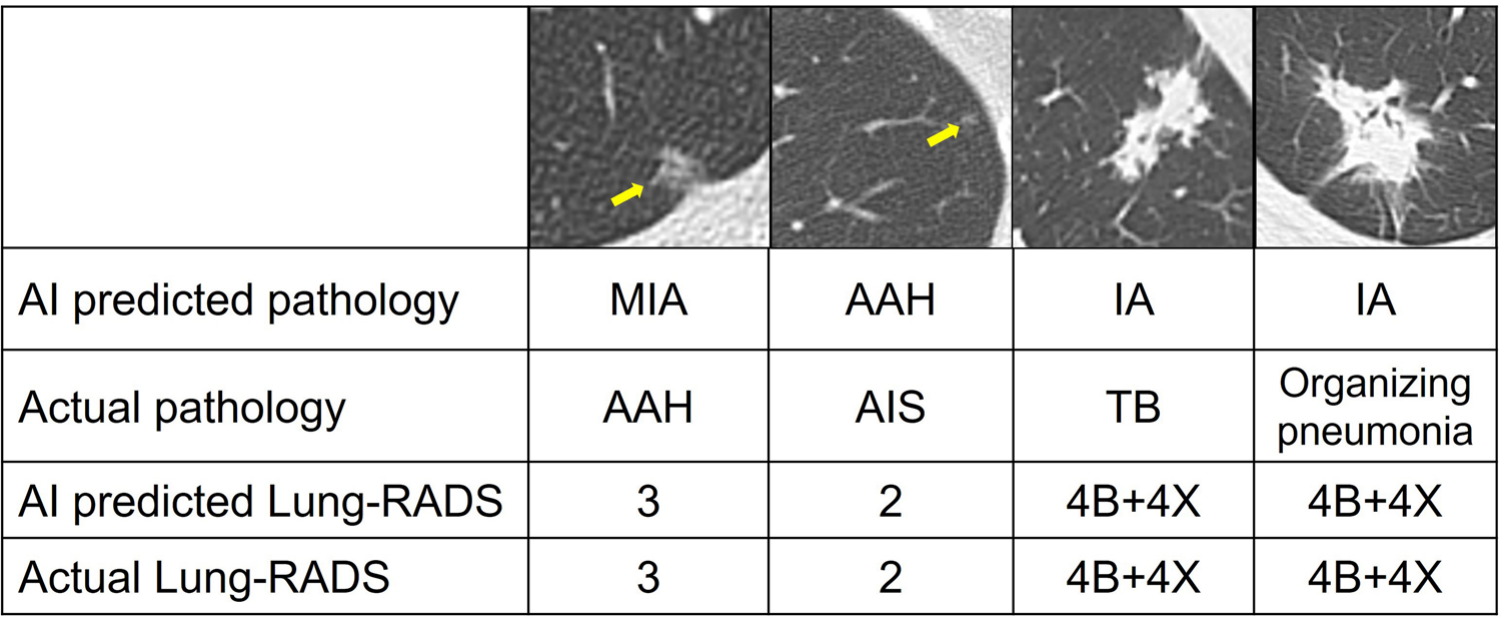
Representations examples of pathology misclassification followed by accurate Lung-RADS categorization using a chest CT AI classification model. **A** Initial misclassification as MIA, later confirmed as AAH upon pathology examination. Correctly classified as Lung-RADS category 3. **B** Initial misclassification as AAH, later confirmed as AIS upon pathology examination. Correctly classified as Lung-RADS category 2. **C** Initial misclassification as IA, later confirmed as TB upon pathology examination. Correctly classified as Lung-RADS category 4B + 4X. **D** Initial misclassification as IA, later confirmed as organizing pneumonia upon pathology examination. Correctly classified as Lung-RADS category 4B + 4X

The result of the ablation study of Task 2.1 shows that clinical data play an important role in the classification of pathological subtypes of lung nodules, increasing the classification accuracy by 1.89%. Smoking has the greatest impact on classification, increasing the accuracy by 0.94% (Table S1). In our study, the LNOP dataset showed a higher risk of malignancy when the Lung-RADS categorization was used as a reference. This may be explained by differences in ethnicity, as Asian populations with adenocarcinoma often presented with ground-glass opacities. This highlights the inadequacy of relying on Lung-RADS alone for follow-up and management decisions. Our AI models could lead to more personalized treatment plans by better predicting the pathological nature of nodules. In cases with incorrect pathological subtype classification but correct Lung-RADS categorization, we found that the inconsistency of solid parts and pathological report is the cause of the suboptimal result of pathological subtype classification (Fig. 6). The spectrum of early-stage lung cancer shows progressive size and density changes on chest CT, making it difficult to accurately differentiate IA, MIA, AIS, and AAH. In Task 2.1, the classification of the AAH/other group has the lowest accuracy because this group also includes other benign lesions, such as tuberculosis (TB), cryptococcal infection, and organizing pneumonia. TB presents with various imaging features, including cavitation, pleural tethering, and spiculation, which can mimic malignancy. As a result, it not only poses a clinical diagnostic problem but also affects the learning effect of the model. Infection and inflammation may appear as ground-glass opacities in the early stages of CT imaging. This can only be confirmed after serial follow-ups and clinical correlations. However, even with these confounding cases in our dataset, the accuracy of predicting IA was 84.70%. After eliminating these confusing benign lesions, the accuracy of IA prediction can increase to 87.33% in Task 2.2 (Fig. 5).

Recent medical literature has demonstrated an emerging trend in the use of DL models for the diagnosis of lung cancer. A literature review of previous studies is summarized in Table 4. Qi et al. achieved an accuracy of 76.9% and a F1 score of 60.9% in a dataset of 448 pure ground glass nodules (GGN), categorizing them as IA or other types [47]. Zhang et al. excelled with an 89.8% accuracy in distinguishing malignant from benign nodules in a dataset of 972 patients after excluding nodules that were difficult to segment [48]. Notably, GGNs are inherently challenging due to their blurred borders, potentially containing malignancies such as AIS. Liu et al. achieved an 81.6% accuracy in a binary malignant versus benign classification in 204 patients [49], while Marappan et al. achieved a 76.67% accuracy in distinguishing MIA from IA in a dataset of 105 patients [50]. Qi et al. extended their study to 417 patients and classified nodules as small cell lung cancer (SCLC), IA, and SqCC. They reported individual accuracies of 0.83, 0.75, and 0.67 for SCLC, IA, and SqCC, respectively, with an average accuracy of 0.75. The weighted F1-average was also 0.75 [51]. Notably, SCLC and SqCC were predominantly observed as pure solid tumors, whereas IA showed a broader distribution from pure GGN to pure solid patterns. Kao et al. focused on pure GGNs in a dataset of 338 patients and achieved an accuracy of 70.6% in differentiating AIS + MIA from IA [52]. These studies used private datasets with specific limitations, including nodule size, solid or ground-glass opacity components, which may impact the applicability of the models. It is well known that the performance of a model can vary across different datasets, making it difficult to generalize results from private datasets to broader clinical scenarios. In this context, we present a quantitative comparison between our private and publicly available datasets, focusing on nodule size and solid component distributions (Fig. 4). This analysis aims to provide a clearer understanding of the differences between our private dataset and public datasets, allowing for an informed assessment of the suitability of the model for clinical practice or research in specific medical domains.

**Table 4:** Comparison with previous studies of model’s performance on private datasets

	Inclusion	Exclusion	Nodule number	Classification	Accuracy	F1-score
Marappan et al. (2022)	Mixed GGN with pathology IA or MIA		105	2-class: IA vs MIA	76.67%	–
Qi et al. (2022)	Pathology: adenocarcinoma, SqCC, SCLC		417	3-class: IA vs SqCC vs SCLC	75.00%	75%
Kao et al. (2022)	Pure GGN with pathology AIS, MIA, or IA	Nodule with solid component; nodule that are not lung cancer	338	2-class: IA vs MIA + AIS	70.60%	–
Qi et al. (2023)	Pure GGN	Nodule with solid component; nodule size < 5 mm or > 30 mm	448	2-class: IA vs MIA + AIS + AAH	76.90%	60.90%
Zhang et al. (2023)	Patient with only one nodule	Hard to segmentation	972	2-class: malignancy vs benign	89.80%	–
Liu et al. (2023)	Patient with only one nodule	COPD, interstitial lung disease, or other diffuse lesion	204	2-class: malignancy vs benign	81.60%	–
Our study (2023)	Nodules with pathology IA, MIA, AIS, AAH, or other benign lesion		963	3-class: IA vs AIS + MIA vs AAH + others	74.76%	75.45%
	Nodules with pathology IA, MIA, AIS, AAH		631	4-class: IA vs MIA vs AIS vs AAH	68.52%	68.70%
	Nodules without pathology		838	4-class: Lung-RADS 2/3/4A/4B + 4X	80.48%	80.38%

CAD has been used for Lung-RADS categorization. Park et al. used CAD to improve the inter-reader agreement of Lung-RADS category, from moderate to substantial with CAD [53]. Nowadays, DL-based CAD can perform automatic nodule detection and automatic Lung-RADS classification. However, it requires radiologists to confirm nodule size, solid part size, and categorization into pure GGN, part-solid nodule, or pure solid nodule step by step before determining the Lung-RADS score. Theoretically, it does not perform Lung-RADS categorization using DL but generates classification results using conditional constructs in its code, without identifying features. For example, in the case of 4X, which refers to category 3 or 4 nodules with additional features or imaging findings that increase the suspicion of malignancy, this classification result relies on the judgment of malignant features. Our model performed better for Lung-RADS scores 2 and 4B + 4X, but is less effective for Lung-RADS scores 3 and 4A. Our model achieved an accuracy of 93.3% in the most severe categorization, 4B + 4X, showing a high degree of consistency with human classification results. The four-class classification had an ordinal severity. This makes it easier to predict the extremes of severity, while the intermediate, ambiguous range is more challenging. Using DL to address the Lung-RADS score classification problem presents several challenges, including the lack of Lung-RADS labeled datasets and the need for a larger dataset for multi-class classification tasks. Ensuring a balanced number of samples for each classification is challenging. As a result, there is relatively little research in this area. In addition, almost all commercial CAD software currently imposes restrictions on nodule sizes, such as 3 mm to 30 mm. However, in our research, we did not limit the nodule size. Therefore, we believe that our model has a broader applicability.

There were some limitations to our study. First, the segmentation of nodules was required for radiomics analysis. Our results showed that radiomics provided additional information apart from that obtained by CNN, indicating that there is potential for improvement in CNN feature extraction. However, with the advent of new tools for automatic or semiautomatic image segmentation, our model could be incorporated into clinical practice in the near future. Second, nodule growth assessment, crucial for Lung-RADS categorization, was not feasible in this study. Our future research will focus on employing AI for the analysis of CT scans obtained at different times to address this issue. Nonetheless, our study provides a promising basis for the development of more accurate and efficient lung nodule classification models.

## Conclusion

In conclusion, our proposed model provides a promising approach for accurately classifying pulmonary nodules based on the benign/malignancy, different pathological subtypes, and Lung-RADS system, which could aid in the diagnosis of lung cancer.

### Supplementary Information

Below is the link to the electronic supplementary material.

Supplementary file1 (DOCX 20 KB)

## Data Availability

All datasets generated for this study are included in the article/supplementary material.

## Abbreviations

Lung-RADS: Lung Imaging Reporting and Data System
LUNA16: Lung Nodule Analysis 2016
LNOP: Lung Nodule Received Operation
LNHE: Lung Nodule in Health Examination
CNN: Convolutional neural network
NLST: National Lung Screening Trial
LDCT: Low-dose computed tomography
MIA: Minimal invasive adenocarcinoma
IA: Invasive adenocarcinoma
CAD: Computer-aided detection
DL: Deep learning
AIS: Adenocarcinoma in situ
AAH: Atypical adenomatous hyperplasia
SqCC: Squamous cell carcinoma
NAS: Neural architectures search
CA: Coordinate attention
CBAM: Convolutional block attention module
DRDB: Dilated residual dense block
GLCM: Gray Level Co-occurrence Matrix
GLRLM: Gray Level Run Length Matrix
GLSZM: Gray Level Size Zone Matrix
GLDM: Gray Level Dependence Matrix
LoG: Laplacian of Gaussian
SE: Squeeze-and-excitation
TB: Tuberculosis
GUI: Graphical user interface
