# Supplementary material for: Combined model integrating deep learning, radiomics, and clinical data to classify lung nodules at chest CT
Source: Radiol Med. 2023 Nov 16;129(1):56–69. doi: 10.1007/s11547-023-01730-6 (PMC10808169; doi:10.1007/s11547-023-01730-6)
Supplement: Supplementary file 1 — Supplementary file1 (DOCX 20 KB) [file 11547_2023_1730_MOESM1_ESM.docx]

**Supplemental Material**

**Appendix S1**

**Training Environment**

The training environment of this study is as follows:

- OS: Windows 10 (64-bit)
- CPU: Intel Core i7-9700H @ 3.00GHz
- Display Card: Single NVIDIA GeForce RTX 2070 - 8GB
- RAM: 24GB
- Deep learning framework: Pytorch

**Training Strategy**

The optimizer and hyperparameters used in this study are as follows:

- Optimizer: Adam with momentum parameters β1 = 0.5 and β2 = 0.999
- Learning rate: 0.0002
- Epoch: 400

The data augmentation method used in this paper is as follows:

- Randomly flipping in XY-plane
- Randomly flipping in XZ-plane
- Randomly flipping in YZ-plane

**Table S1**

**Supplementary Table 1** Ablation tests on integrating clinical features on the performance of malignancy prediction.

| **DL + radiomics features** | **Clinical features** | | | | **Accuracy (%)** | **F1-score (%)** | | | |
| --- | --- | --- | --- | --- | --- | --- | --- | --- | --- |
|  | **Smoking** | **Family history** | **Age** | **Sex** |  | **Macro averaged** | **Others/AAH** | **AIS/MIA** | **IA** |
| ✔ |  |  |  |  | 72.87 | 73.57 | 70.15 | 70.83 | 78.97 |
| ✔ | ✔ |  |  |  | 73.81 | 74.51 | 71.29 | 71.95 | 79.45 |
| ✔ | ✔ | ✔ |  |  | 74.02 | 74.77 | 71.42 | 72.11 | 79.59 |
| ✔ | ✔ | ✔ | ✔ |  | 74.44 | 75.10 | **72.16** | 72.05 | 80.51 |
| ✔ | ✔ | ✔ | ✔ | ✔ | **74.76** | **75.45** | 71.52 | **72.76** | **81.49** |

**Table S2**

**Supplementary Table 2** The relationship between final pathology and Lung-RADS score in the LNOP dataset.

| **Lung-RADS score**  **Pathology** | **2** | **3** | **4A** | **4B+4X** |
| --- | --- | --- | --- | --- |
| **Others + AAH** | 60.2% | 29.1% | 22.7% | 20.0% |
| **AIS + MIA** | 39.8% | 58.9% | 29.9% | 3.2% |
| **IA** | 0.0% | 12.0% | 47.4% | 76.8% |
